# Extrachromosomal Circular DNA and Transposable Elements in Type 2 Diabetes

**DOI:** 10.3390/ijms262311516

**Published:** 2025-11-27

**Authors:** Celeste Moya-Valera, Alex Fernando Arita, Francisco Lara-Hernández, Ana-Bárbara García-García, Felipe Javier Chaves

**Affiliations:** 1 Genomics and Diabetes Unit, INCLIVA Biomedical Research Institute, 46010 Valencia, Spain; cmoya@incliva.es (C.M.-V.); franlh19@gmail.com (F.L.-H.); felipe.chaves@uv.es (F.J.C.); 2 CIBERDEM, ISCIII, 28029 Madrid, Spain

**Keywords:** extrachromosomal circular DNA, transposable elements, type 2 diabetes, mechanisms

## Abstract

Type 2 Diabetes (T2D) is a complex disease that arises from interaction between genetic and environmental factors. The activation of transposable elements (TEs) and the production of circular extrachromosomal DNA (eccDNA) may represent genetic mechanisms involved in cellular aging, metabolic alterations, and T2D development through distinct pathways. Although the origin and characteristics of TEs and eccDNA differ substantially, eccDNA can in some cases be derived from TEs. This review summarizes the current understanding of these mechanisms and examines the reported associations between T2D and either TEs or eccDNAs. These findings highlight the significant involvement of these molecules in disease pathogenesis, particularly in relation to aging, and underscore their great potential as biomarkers and targets for T2D prevention.

## 1. Introduction

Type 2 diabetes (T2D) accounts for 90–95% of all diabetes cases and is characterized by elevated glucose levels resulting from impaired insulin action and/or secretion [1]. Its prevalence has increased markedly, from 320 million individuals in 2010 to 530 million in 2021, and is projected to exceed 1.3 billion by 2050 [2]. In Spain, approximately 13.5% of adults over 18 are affected by T2D, a prevalence rising sharply with age, reaching nearly twice the rates observed in younger populations [3,4]. T2D has a significant impact on mortality, morbidity, and healthcare costs [2]. It is a complex disease influenced by multiple factors. Genetic studies in younger, non-obese individuals aim to identify risk factors associated with increased genetic load. Aging is another major driver of T2D development, acting through diverse biological mechanisms [5], and underscores the importance of investigating its molecular underpinnings.

Research into T2D therefore focuses on both genetic and non-genetic factors, the former encompassing genetic variation and epigenetic marks, while the latter include body mass index (BMI), age, sex, nutrition, and physical activity [6]. These factors also interact with each other, thus increasing complexity, and environmental factors such as metal concentration and oxidative stress (OS) can modulate the effect of many genetic variants in relation to T2D or its main risk factors [7,8,9]. To date, over 700 loci associated with T2D have been identified in the literature or in databases, a number that represents less than 20% of the overall estimated genetic component [10,11,12]. Furthermore, the different organs and cell types involved in T2D can be affected differently. For example, hepatocytes develop metabolic alterations leading to insulin resistance (IR), whereas pancreatic beta cells increase insulin production to compensate for IR, causing cellular stress, malfunction, reduced insulin secretion and/or cellular death [13]. In addition, other insulin-sensitive tissues, such as adipose tissue or muscle, have an important role in the development of inflammation, IR and T2D, particularly in older individuals [14,15,16].

The major risk factors contributing to T2D development include older age, high BMI, IR, oxidative stress (OS), and inflammation. These factors are closely linked to genetic and genomic mechanisms that may contribute to metabolic alterations involved in T2D progression, yet the specific related genomic mechanisms implicated in T2D remain unidentified. Along with genetic variations [10,11,12], two mechanisms potentially involved in T2D development are circular extrachromosomal DNA (eccDNA) generation and the activity/deregulation of transposable elements (TEs). Both processes can exert deleterious effects, are more active with aging, and are associated with age-related health complications [17,18,19]. These mechanisms are interconnected, as TEs can form part of eccDNAs and facilitate their generation [20,21,22]. Nonetheless, little is known about the role of TEs and eccDNAs in T2D. This review summarizes key findings regarding these two mechanisms, highlighting knowledge gaps that warrant further investigation.

## 2. Extrachromosomal Circular DNAs

EccDNA was first described by Hotta in 1964 [23] and detected in aging cells and premature aging disease in 1985 [24]. It has recently attracted renewed attention due to advances in detection technologies and its emerging role in diverse human processes and diseases [19,25,26]. EccDNAs are circular DNA molecules derived from all chromosomes and represent a major component of extrachromosomal DNA. Specific DNA regions can be protected from eccDNA generation, while others may facilitate it [27,28]. EccDNA levels in cells depend on different factors including their formation, replication, segregation, elimination and selection [28]. In the extracellular media, eccDNA levels depend on other factors such as the eccDNA levels inside cells, their secretion and cellular death, and eccDNA elimination from this media by degradation by nucleases or cellular uptake. EccDNA molecules vary widely in size, ranging from hundreds to millions of base pairs (bp), and can be categorized based on their genomic origin, size, and sequence. Based on these features, eccDNAs are categorized into extrachromosomal DNA (ecDNA) or double minutes (DMs; ~100 kb–several Mb), small polydispersed circular DNA (spcDNA; ~100 bp–10 kb), microDNA (~100–400 bp), and telomeric circles (t-circles; multiples of 738 bp) [26,28,29,30]. In addition, complex eccDNA can be formed from preexisting eccDNA, from DNA fragments or by eccDNA mutation [28].

### 2.1. Extrachromosomal Circular DNA Generation and Elimination

Multiple mechanisms contribute to the generation of eccDNAs, including breakage-fusion-bridge, chromothripsis, translocation-deletion-amplification, and regions associated with high transcription activity. The primary mechanisms involved in eccDNA formation include homologous and non-homologous recombination, DNA replication, and the formation of R-loops [29,31]. The recurrent presence of specific eccDNAs carrying functional genes in different individuals suggests that eccDNA formation is not random but often involves recurrent genomic sequences [25]. In addition, the correlation between the number of eccDNAs per Mb and protein-coding genes per Mb and the increased number of *Alu* sequences in regions producing eccDNA suggests that eccDNA generation may be associated with the openness of chromatin and transcription activity [32,33,34].

EccDNA generation is frequently associated with cellular stress induced by aging, OS, inflammation, and apoptosis, among other factors. For instance, GSTM2 reduces eccDNA production by lowering DNA damage and, therefore, reduces interferon I-stimulated inflammation in cardiac tissue [35]. Conversely, eccDNA can activate inflammatory response by different tissue-specific pathways and at the systemic level via circulating eccDNA [35,36,37]. These inflammatory responses affect mitochondrial function, increasing levels of OS, apoptosis and DNA damage, thereby contributing to aging and associated diseases, including T2D [38].

Although eccDNAs have been linked to various diseases, they are also widely present in healthy somatic cells in humans [19,25,36,39,40]. Their abundance and composition vary considerably across cell types, are influenced by stress and disease states, and generally show an age-dependent increase [17,18,26,41,42,43]. EccDNA regulates mRNA levels by different mechanisms and some studies have found a relationship between them in different tissues, including some related to T2D [27,44]. EccDNA generation can facilitate the movement of TEs, as some eccDNAs are themselves derived from TEs, and certain TEs are involved in eccDNA generation and can promote their production [45].

In adipose stem cells, results indicate that cells from young donors have higher eccDNA levels than those from older donors, and some eccDNAs are lost with aging. This finding may appear contradictory to previous reports [32]. These differences could be related to the differentiation capacity of stem cells and a possible role of eccDNA [34]. Recent data indicates that eccDNA production per unit of transcription is increased, although the total number of eccDNA can be reduced due to lower transcription levels, and that eccDNA profiles are tissue-specific [27]. These data suggest that specific groups of eccDNA may follow different patterns in stem cells compared to other somatic cells.

EccDNAs encompass diverse genomic elements, including genes, regulatory regions, intergenic regions, telomeres, centromeres, high complexity regions, and TEs [25,46]. Some studies have identified eccDNAs containing complete genes that enhance cellular response to environmental stimuli, particularly in cancer cells [40]. In addition, the high abundance of eccDNAs in somatic genomes suggests that they may shape cellular phenotypes by altering gene copy numbers and regulating the transcription of full-length or truncated genes [25,29]. In this way, eccDNAs may contribute to cell development, aging, adaptive evolution, and tumor progression by affecting signaling pathways, telomere length regulation, genome plasticity, sequestration of transcription factors, and gene amplification [17,29].

EccDNA generation is recognized as one of the mechanisms involved in cellular adaptation and clonal selection [29,47]. Genetic alterations resulting from eccDNA, such as deletions or amplifications of specific genomic regions, can persist in certain cells and their progeny. If these alterations confer a selective advantage, they can facilitate cellular adaptation and clonal selection, particularly in aging cells [29,39,47]. The primary contribution of eccDNAs to this phenomenon may be their preferential formation in genes exhibiting high transcription rates under specific conditions [39].

Elimination of eccDNA is an important factor in the regulation of its own levels but there is limited information on this subject. Some studies have shown that cell-free eccDNAs present in plasma are degraded by DNASE1L3 while DNASE1 has no effect, and that neither DNASE1L3 nor DNASE1 affects intracellular eccDNA [48]. In systemic lupus erythematosus, the loss of DNASE1L3 activity generates specific eccDNA profiles and the presence of eccDNA from specific genes and specific disease symptoms [49,50]. Intracellularly, the elimination of specific eccDNAs has been described through micronuclei formation and secretion as well as reintegration into the genome [29,51,52]. This process can be increased by DNA damage or transcription repression of eccDNAs [28]. In young and healthy cells, eccDNAs are generally directed via nuclear actin towards the nuclear pore complex for exclusion from the nucleus. In aging and age-related disorders, a reduction of this mechanism causes the accumulation of eccDNA in the nucleus. Therefore, in aging and age-related diseases, malfunction of actin rods and nuclear pores decreases eccDNA exclusion from the nucleus, which may contribute to cellular malfunction [18,53].

### 2.2. Extrachromosomal Circular DNA Roles and Modulation

EccDNA functions are strongly influenced by their size and sequence composition, which ultimately determine their biological roles. Large eccDNA can harbor intact genes, modulate transcription, and increase chromatin accessibility, thereby enhancing gene expression [54]. In contrast, small eccDNA molecules may regulate transcription by sequestering transcription factors, contributing to genome instability, and playing a role in the generation of chromosomal rearrangements. They can also arise as by-products of DNA damage, particularly during apoptosis [17,36]. In this way, cell type-specific eccDNAs can be related to T2D development and progression as they can modulate cellular metabolisms in fat, liver, kidney and pancreas, the main players in glucose metabolism [27,44,55].

The results from Liang et al. show that eccDNA variability increases with age in the different tissues and provide evidence that the number of eccDNA per gene is affected by the level of transcription and that the eccDNA profiles are thereby tissue-specific [27]. In pancreatic beta cells, eccDNA patterns change with the development of T2D and become enriched in the glucagon signaling pathway, while, in later stages of T2D, the eccDNAs are enriched in the phosphatidylinositol signaling system, both related to metabolic regulation [56,57,58].

Within cells, eccDNAs are primarily localized in the nucleus but can also be exported to the cytoplasm for degradation. Moreover, they are detectable as cell-free DNA in biofluids such as blood and urine, exhibiting variations between cases and controls, likely due to apoptosis across different cell types [17,59]. While many studies have investigated eccDNAs in the context of cancer, where they play significant roles [19], emerging research suggests that small eccDNAs originating from apoptotic processes may exert notable effects on inflammation by stimulating IFNB and interferon responses [18,36]. Furthermore, eccDNAs have been associated with age-related diseases, including chronic inflammation in senescent cells and premature aging, such as Werner syndrome [24,60].

### 2.3. Extrachromosomal Circular DNA and Type 2 Diabetes: Direct Evidence

Although eccDNA has been associated with the aging process, only a limited number of studies have examined eccDNA in the context of T2D. Evidence suggests that eccDNA levels rise in response to intensive insulin treatment among T2D patients, particularly for eccDNA derived from repetitive DNA, with differential gene presence observed between pre- and post-treatment, mainly in inflammation and metabolic genes [35]. Kong et al. reported that newly diagnosed T2D patients display enrichment of multiple eccDNAs, including a specific molecule, SORBS1^circle^, which is elevated in these patients and linked to IR both in vivo and in HepG2 cells under induced IR conditions [61]. Notably, SORBS1^circle^ was also associated with apoptotic DNA fragmentation in HepG2 cells [61]. Altered eccDNA production has also been reported in women who subsequently develop gestational diabetes, with 2217 eccDNAs differentially detected, including reduced PRDM16^circle^ levels [62].

Both *SORBS1* and *PRDM16* have been associated with T2D risk [63,64]. *SORBS1* is a key gene controlling glucose uptake in muscle and adipocytes and plays a role in regulating IR [65,66]. *SORBS1* is also involved in mitochondrial respiration through complex I assembly, an essential component of glucose metabolism and diabetic pathogenesis and a therapeutic target influencing treatment response [67,68,69].

PRDM16 may modulate glucose metabolism as it is a transcriptional regulator that interacts with PPARg, C/EBPb or PGC-1 to modulate brown fat differentiation, body weight, glucose and lipid metabolism, and energy homeostasis. In addition, mice overexpressing *PRDM16* in adipose tissue display markedly increased insulin sensitivity and energy expenditure in response to a high-fat diet [63]. Elevated eccDNA levels could be partly related to increased DNA breaks in T2D patients due to cellular stress caused by high glucose levels. Following treatment, the reduction in eccDNA corresponds with the normalization of glucose [35,70,71].

### 2.4. Extrachromosomal Circular DNA and Type 2 Diabetes: Indirect Evidence

EccDNAs show changes linked to T2D risk factors such as sedentary lifestyle or aging and T2D complications [43,59,72,73]. Gerovska et al. [72] reported that most of the genes showing significant differences in eccDNA levels in muscle tissue from sedentary and active patients, such as *AGBL4*, *RNF213*, *MED13*, *WWTR1*, *ZBTB7C*, *ITPR2*, *DDX11-AS1* and *RYR2*, are linked to diabetes, glucose and lipid metabolisms and cellular senescence [36,74,75,76,77,78,79,80].

T2D is known to be an inflammatory disease [81,82] and eccDNAs have a key role in the immune response [36]. EccDNAs can activate the innate immune response and the production of different proinflammatory cytokines, leading to a potent immunostimulatory activity that may induce primary B-cell and T-helper cell responses [36,53,82].

Furthermore, eccDNA production is increased in the male germline cells of diabetic patients, which could have substantial transient and permanent effects on germ cells, as eccDNA can be expressed and reintegrated into the genome [83,84].

Collectively, these findings suggest a potential association between T2D and eccDNA generation, likely driven by diabetes-induced genomic damage and cell death. In turn, cellular alterations induced by eccDNAs can contribute to the development of T2D and would be good biomarkers for the disease. Specific eccDNAs, such as SORBS1^circle^ and PRDM16^circle^, can be used as biomarkers for T2D, but many others, including genes regulating glucose metabolism or genes related to tissues damaged by high glucose or drug metabolism, could be interesting to predict organ damage and treatment response. In addition, other eccDNAs (comprising genes or regulatory sequences related to IR, damage to pancreatic beta cells or metabolic alterations preceding T2D) could be used in T2D risk estimation.

Table 1 shows a scheme with relationships between eccDNAs and T2D.

## 3. Transposable Elements

The second mechanism related to DNA integrity and stability that may be linked to T2D involves the activity and mobility of TEs, which are DNA sequences that can move from one site to another in the genome. Several publications over the years have reviewed their classification systems and several tools are used for their classification and annotation [85,86,87,88,89,90]. TEs were first discovered in 1944 by McClintock as “controlling elements” [91] and were later described as repetitive sequences and genetic elements capable of transposing between genomic locations [88,92,93].

TEs represent a significant portion of most genomes. In humans, they account for more than 45% of the genome, with non-long terminal repeat (non-LTR) retrotransposons comprising ~75%, LTR retrotransposons ~20%, and DNA transposons ~6%. Nevertheless, most of these elements are currently inactive [43].

Retrotransposons, including LTR and non-LTR retrotransposons, undergo a process involving transcription, reverse transcription, and insertion into the genome, facilitated by their own reverse transcriptase enzyme or by enzymes encoded by other TEs. Conversely, DNA transposons employ a cut-and-paste mechanism for self-propagation [94]. Active TEs produce various types of RNA molecules, with mRNAs being the most common [95].

TEs play major roles in genome evolution through various mechanisms such as insertion, DNA recombination, chromosomal rearrangements, mutation, gene expression regulation, and epigenetic alterations. These mechanisms also contribute to the development of human diseases including cancer and various genetic disorders. TEs are now recognized as essential components in genome regulation and structure, influencing development, health, disease, environmental responses, and aging [43,96]. TE insertions can have diverse effects on affected genes or genetic regions, including gene inactivation through mutation, insertion of sequences into RNA, splicing alteration, transcriptional changes, and eccDNA generation [20,22,47,95]. To counteract these deleterious effects, TE transcription is suppressed in cells through mechanisms such as DNA methylation (DNAm), histone signaling guided by small non-coding RNAs (mainly piwiRNAs), and various protein complexes. These systems help cells to regulate and contain TEs through repression [97]. TEs are also regulated by transcriptional and posttranscriptional mechanisms. At the transcriptional level, stress-induced formation of ribonucleoprotein granules (stress granules and processing bodies) may prevent transposition [98]. At the post-transcriptional level, RNA degradation, adenine methylation, RNA 3′-end polyadenylation and uridylation may occur [99].

However, the effectiveness of these systems diminishes with age, cancer and age-related diseases, such as T2D [41,42,43,100]. In addition, the innate immune system can recognize DNA and RNA from TEs, increasing inflammation and leading to their degradation, thereby contributing to the regulation of DNA and RNA levels. This inflammation is mediated by different pathways but primarily through Type I IFN response [42,101,102]. Furthermore, mutations in the *IFIH1* gene, which is involved in nucleic acid sensing, have been associated with autoimmune diseases, Type 1 Diabetes and Aicardi–Goutières syndrome [101,103,104].

Situations that increase cellular stress and/or modify DNAm, including aging, OS, exposure to environmental contaminants, and inflammation, can modulate TE expression [43,105,106,107], although certain TEs may be involved in stress response [108]. Notably, T2D is often accompanied by OS, inflammation, and alterations in DNAm, which may serve as initial hallmarks of the processes leading to T2D development [109,110]. Other research has also identified DNAm alterations preceding T2D onset [111,112], although these studies typically do not specifically analyze DNAm in TE regions. Exposure to high blood glucose levels induces DNA methylation changes that can persist after glucose normalization for long periods, including in subsequent generations, by epigenetic mechanisms [113,114]. The effects of early life nutrition on DNA methylation and TEs can have a major influence on metabolism in adulthood and subsequent generations [114,115]. Some studies have identified alterations in DNAm within LINE-1 elements in T2D [116].

### 3.1. Transposable Elements and Type 2 Diabetes: Direct Evidence

Some authors suggest a possible link between TEs and T2D [42] as the RNA levels of different mobile elements increase with aging and senescence in various tissues such as muscle and liver [41,117]. Furthermore, both T2D and a high-fat diet have been found to increase the expression of *Alu* elements while reducing DICER1 levels (a protein responsible for breaking down *Alu* mRNA), causing DNA damage [102,118]. Decreased *Alu* methylation has been associated with increased HbA1c levels [119] and global DNAm in LINE1 has been associated with the metabolic status of T2D patients [116,120]. A review including 14 observational and 6 interventional studies concluded that *LINE-1* methylation correlated with body composition and obesity-related disorders, including T2D, insulin resistance, and CVD [121]. In addition, genome-wide DNAm analysis can provide relevant information about methylation alterations in specific TEs and their relationships with T2D and its consequences.

An interesting link between TEs and T2D is the protective effect of nucleoside reverse transcriptase inhibitors (NRTIs) against T2D and their ability to improve IR. This effect may be explained by the important role of the inflammasome in T2D, its activation by *Alu* RNA sequences, and the reduction of *Alu* sequence propagation along with the normalization of DICER1 activity by NRTIs [42,102]. Additionally, NRTI inhibition of reverse transcriptases from viruses and retrotransposons is directly linked to the prevention of T2D by reducing the deleterious effect of retrotransposon movement. NRTIs appear to protect against T2D, whereas other reverse transcriptase inhibitors may damage pancreatic beta cells and increase the risk of T2D mediated by OS and mitochondrial toxicity [122].

Notably, PPARs (nuclear hormone receptors that act as transcription factors in response to endogenous lipid messengers) have been identified as modifiers of TE expression. Their agonists, which are used in the treatment of T2D and dyslipidemia, can act as modifiers of TE expression levels, although whether this effect contributes to therapeutic benefit has not yet been elucidated [123].

On the other hand, it is also known that fatty liver disease and other liver metabolic alterations are risk factors for T2D development [124]. In this regard, alterations of TEs activity and liver metabolic regulation have been described, including changes in DNAm [113,125,126].

With respect to genetic variations, many polymorphic TEs have been identified in loci or genes related to complex diseases, metabolic alterations and response to environmental factors that can modulate T2D development [43,127].

### 3.2. Transposable Elements and Type 2 Diabetes: Indirect Evidence

There is indirect evidence linking TEs to T2D, such as the capacity of cell-free DNA (cfDNA) and eccDNA, both containing TEs, to integrate randomly into the genome of exposed cells, leading to cellular damage, stress, and apoptosis, likely due to the TEs present in both [128]. Situations that increase TEs activation in a cell can therefore induce cellular damage in other cells and organs, promoting aging and increasing the risk of associated diseases such as T2D.

In addition, increased levels of TEs activity activate proinflammatory systems [42,101,102], and, as indicated above, T2D is an inflammatory disease [81,82]. TEs have a relevant role in multiple processes through a complex RNA regulatory network and transcriptional regulatory functions (including transcriptome modulation and cellular identity establishment). They contribute to the regulation of cellular plasticity and adaptability to environmental cues through their RNAs [129]. Regarding the immune system, TEs are involved in its development, activation, over-activation, response to different factors and regulation of gene expression [130,131,132].

Another link between TEs and T2D is the role of the ZFP92 transcription factor. ZFP92 binds to TEs in pancreatic beta cells, muscle and adipocytes, repressing them while activating specific genes. This activity regulates beta cell development and lipolysis in adipocytes and muscle, ultimately modulating glucose metabolism in mice [133]. Finally, PiwiRNAs, potent inhibitors of transposon activity, may also play a role in pancreatic beta cell function and metabolic regulation [134]. However, these RNAs have many other functions, and there is no direct evidence of their role in TEs in this context.

Different TEs can be interesting biomarkers in T2D, mainly those that remain active or can move in the genome as *Alu* or LINE1 elements. Other markers can be derived from their own activity as transcription or the transposition rate in specific cells or tissues. Finally, polymorphic TEs may be of interest as biomarkers.

Table 2 summarizes the described associations between TEs and T2D.

## 4. EccDNA and TEs in Organ Damage Related to T2D

T2D generates different kinds of organ damage including diabetic retinopathy, neuropathy, kidney disease, and diabetic cardiovascular disorders [135,136]. The basis of these complications has been analyzed from genetic, epigenetic and metabolic points of view, but there is little information about the possible role of eccDNA or TEs [137,138,139]. It is interesting to note that eccDNA generation and TEs expression differ between tissues in adulthood and during development and they regulate tissue-specific gene expression [41,84].

As previously indicated, TEs and eccDNA have been involved in inflammation at the systemic level and in different organs; therefore, part of their effect on organ damage can be explained by this relationship [89,107]. In this context, TEs have been associated with brain development, neurological diseases [107,140], and altered DNAm of LINE1, and *Alu* sequences have been found in T2D patients with pre-symptomatic dementia [141]. In renal disease, TEs, especially endogenous retroviruses, have been shown to increase inflammation in the kidney and have been implicated in kidney disease development in T2D [142]. In addition, T2D patients with cataracts show hypomethylation in *Alu* and LINE1 sequences compared with healthy controls and diabetic patients without cataracts [143]. Finally, Sabbatinelli et al. found a differentiated methylation region related to mortality of T2D patients that overlaps *TIGD3* (Tigger-transposable element derived 3), a gene encoding a DNA-transposable element [144].

## 5. Conclusions

Future Directions: previous data indicate the potential use of eccDNAs and TEs as valuable biomarkers for predicting IR, diabetes and treatment response. In the near future, additional biomarkers related to TE and eccDNA may be identified, including DNAm alterations in specific TEs, mRNA, peptides or proteins derived from both, as well as DICER activity or DNAse activity. Further studies are required to identify biomarkers that are more specific for disease prediction, treatment response, and the presence or progression of organ damage.

Moreover, studying eccDNA may help uncover new genes involved in T2D and its associated complications. Different strategies for T2D prevention and treatment could also be developed in relation to eccDNA and TEs, for instance, by reducing TE expression, promoting degradation of their RNAs or decreasing related inflammation, as seen with the use of NRTIs. Finally, both types of molecules could potentially be harnessed in T2D therapy for the targeted introduction of specific genes into cells to modulate their metabolic activity.

**Figure 1:**
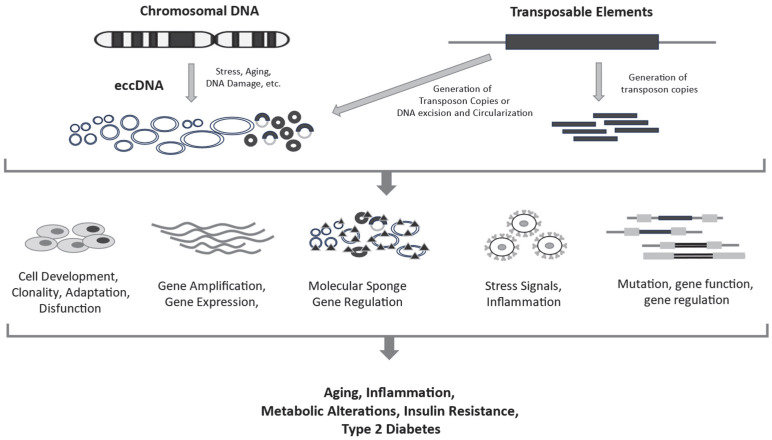
Summary of associations between eccDNAs, TEs and T2D. Both eccDNAs and TEs are involved in processes related to aging, inflammation, metabolic alterations, IR and T2D.

The available evidence suggests that eccDNAs and TEs may contribute to inflammation, aging and metabolism alterations and T2D; however, they have not been directly linked to T2D development (Figure 1). Different stimuli, such as OS, hyperglycemia or other stressful conditions present in the early stages of metabolic alterations leading to T2D, can induce an increase in eccDNA production and TE expression, thereby facilitating T2D development. In addition, increased activity of both systems could play a role in organ damage associated with T2D. Overall, these mechanisms are particularly significant in older individuals, where genome deregulation is heightened and eccDNA and TEs are elevated. While minor or localized DNA damage may be repaired without major short-term effects, repeated DNA damage or impaired repair capacity can modulate metabolism, OS, inflammation and other processes related to IR and T2D development. If this condition persists, it may further enhance genome alteration through eccDNA production and TE activation, driving the cells into a feedback loop that promotes metabolic alterations and T2D. These situations, characterized by increased DNA damage and impaired DNA repair, are more likely to occur with aging.

In conclusion, accumulating evidence supports the potential links between the alterations in eccDNA levels and TE activity, as both are possible causes and consequences of T2D, particularly in the context of aging. The observed correlations, their relationships with T2D risk factors, and the indirect evidence of their involvement underscore the need for further investigation into their mechanistic roles in T2D pathogenesis.

## Figures and Tables

**Table 1:** Relationships between eccDNAs and T2D.

Described Association	Cited in
EccDNAs activate inflammatory response in specific tissues and in whole organisms: affect mitochondrial function; increase levels of OS, apoptosis, and DNA damage; and accelerate aging and associated diseases, including T2D.	[35,36,37,38]
EccDNAs regulate mRNA levels in different tissues including those related to T2D.	[27,44]
EccDNA generation can facilitate the movement of TEs.	[45]
The high abundance of eccDNAs in somatic cells suggests their influence on cellular phenotypes.	[25,29]
EccDNAs contribute to cell adaptation and clonal selection including roles in cellular development, aging, adaptive evolution, signalling pathways, telomere length regulation, genome plasticity, sequestration of transcription factors, and gene amplification.	[17,29,47]
Large eccDNAs can modulate transcription by increasing chromatin accessibility. Small eccDNAs may regulate transcription by sequestering transcription factors. EccDNA contributes to genome instability and plays a role in the generation of chromosomal rearrangements.	[54]
Cell type-specific eccDNAs can be related to T2D development and pro-gression as they can modulate gene expression in tissues related to insulin signaling, such as adipose tissues, liver, kidney and pancreas.	[27,32,44,55]
Small eccDNAs originating from apoptotic processes may exert notable effects on inflammation by stimulating IFNB and interferon responses.	[18,36]
Newly diagnosed T2D patients display enrichment of multiple eccDNAs, including specific molecules such as SORBS1^circle^.	[58]
EccDNA production is different in women who develop gestational diabetes compared with those with normal glucose metabolism.	[59]
EccDNAs show changes linked to T2D risk factors such as sedentarism or aging and T2D complications.	[43,56,69,70]
EccDNA activates the innate immune response and the production of different pro-inflammatory cytokines.	[36,53,79]
EccDNA production is increased in male germline cells of diabetic patients.	[80,81]

**Table 2:** Relationships between TEs and T2D.

Described Association	Cited in
TE activation in aging: increase in RNA levels from TEs	[41,42,114]
T2D and a high-fat diet increase *Alu* element expression and reduce DICER1 levels	[99,115]
TEs present in cfDNA can integrate randomly into the genome: cellular damage, stress, and apoptosis	[125]
TEs present in cfDNA can integrate randomly into the genome: TE activation in a cell or organ can induce cellular damage in other cells and organs	[125]
Increased levels of TE activity activate the proinflammatory system	[42,98,99]
TEs have a relevant role in transcriptional regulatory functions: regulation of cellular plasticity and adaptability to environmental cues	[126]
TEs are involved in immune system development, activation, over-activation and response to different factors	[127,128,129]
Polymorphic TEs have been related to complex diseases, metabolic alterations and response to environmental factors related to T2D development	[43,124]
TEs are involved in fatty liver disease and other liver alterations related to T2D	[122,123]
ZFP92 transcription factor binds to TEs in pancreatic beta cells, muscle and adipocytes, repressing them and activating specific genes involved in their development and metabolic regulation.	[130]
NRTIs protect against T2D and improve IR by reducing *Alu* copies and DICER1 activity normalization: retrotransposon movement reduction	[42,99]
*Alu* and LINE1 methylation is associated with the metabolic status of T2D patients.	[113,116,117]
Agonists of PPARs can modify TE expression	[120]

## Data Availability

No data were generated in this work.
